# Definition and validation of operating equations for poly(vinyl alcohol)-poly(lactide-co-glycolide) microfiltration membrane-scaffold bioreactors

**DOI:** 10.1002/bit.22815

**Published:** 2010-10-01

**Authors:** RJ Shipley, SL Waters, MJ Ellis

**Affiliations:** 1 Oxford Centre for Industrial and Applied Mathematics, Mathematical Institute24-29 St. Giles', Oxford OX1 3LB, UK; 2 Department of Chemical Engineering, University of BathBath BA2 7AY, UK

**Keywords:** tissue engineering, bioreactor, microfiltration, permeability slip, mathematical modeling

## Abstract

The aim of this work is to provide operating data for biodegradable hollow fiber membrane bioreactors. The physicochemical cell culture environment can be controlled with the permeate flowrate, so this aim necessitates the provision of operating equations that enable end-users to set the pressures and feed flowrates to obtain their desired culture environment. In this paper, theoretical expressions for the pure water retentate and permeate flowrates, derived using lubrication theory, are compared against experimental data for a single fiber poly(vinyl alcohol)–poly(lactide-co-glycolide) crossflow module to give values for the membrane permeability and slip. Analysis of the width of the boundary layer region where slip effects are important, together with the sensitivity of the retentate and permeate equations to the slip parameter, show that slip is insignificant for these membranes, which have a mean pore diameter of 1.1 µm. The experimental data is used to determine a membrane permeability, of *k* = 1.86 × 10^−16^ m^2^, and to validate the model. It was concluded that the operating equation that relates the permeate to feed ratio, *c*, lumen inlet flowrate, *Q*_*l*,in_, lumen outlet pressure, *P*_1_, and ECS outlet pressure, *P*_0_, is
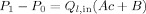
1
where *A* and *B* are constants that depend on the membrane permeability and geometry (and are given explicitly). Finally, two worked examples are presented to demonstrate how a tissue engineer can use Equation 1 to specify operating conditions for their bioreactor.

## Introduction

Tissue engineering shows great promise in regenerative medicine, as it has the potential to solve problems of limited donor grafts for tissue and organ repair, particularly for an aging population. A significant challenge in the field is to transfer lab-scale experimental work to a clinical scale (Dawson and Oreffo, 2008; Thomas et al., 2007, i.e., a cell population in the region of 10^8^ cells and a construct with overall dimensions to the order of centimetres by tens of centimetres), whereby advances in the field can be exploited for clinical benefit to patients.

In hollow fiber membrane bioreactors (HFB), cells can be seeded on the outer surface of the fibers (the method used by the authors), or in a matrix surrounding the fibers Ye et al. (2006), and media is driven through the fiber lumen under an applied pressure gradient. The membrane wall is porous, and so nutrients and proteins (such as growth factors) can permeate across it to the cells. The membrane protects the cells from the direct effect of shear so that relatively high-feed rates can be used without cell damage. Knazek et al. (1972) were the first to report using a HFB for mammalian cell culture; since then the use of HFBs for mammalian cell expansion has become well-documented by Tharakan and Chau (1986), and several cell types have been cultured in HFBs including lymphocytes (Gloeckner and Lemke, 2001; Gramer and Poeschl, 2000) and hepatocytes (Nyberg et al. 1994).

HFB are ideal for tissue engineering on a clinical scale because of the large surface area to volume ratio will reduce the requirements of reagents, labor, and space: a hollow fiber system can be used to culture the same number of cells in 0.5 L as 1 m^3^ using standard flask culture techniques (Ellis et al., 2005) and large cell numbers of up to 2 × 10^8^ cell/mL can be obtained (Scragg, 1991). The fiber lumen mimics a capillary in that it supplies nutrients in vitro, and it is envisaged that this structure will guide angiogenesis in vivo. The issue of scale-up is relatively simple for HFB and since the cells are separated from the bulk flow of media, the flowrate can be set based on the required transport across the membrane without the usual constraint of shear stress on the cells. Fabricating the hollow fiber membranes from a biodegradable polymer such as poly(lactide-co-glycolide) (PLGA) has the potential to allow complete regeneration of the tissue because the scaffold will breakdown as new tissue grows, eventually leaving just the new tissue.

We have previously developed biodegradable PLGA hollow fiber membrane scaffolds (the scaffold being a single fiber or bundle of fibers), for tissue engineering applications by Ellis and Chaudhuri (2007, 2008) and Morgan et al. (2007). PLGA is a common tissue engineering material but the material poses some challenges when compared to established membrane technology. For example, PLGA is biodegradable, so the physical properties of the membrane will change over longer culture periods (weeks–months). Additionally, PLGA is a hydrophobic material and is highly sensitive to chemical treatment. This eliminates the use of standard wetting agents such as ethanol, which cause deformation and fusion of fibers that are in contact Shearer et al. (2006). More recently, we have developed a blended poly(vinyl alcohol)–PLGA (PVA–PLGA) hollow fiber to improve the hydrophilicity of the membranes; this allows wetting without any agents, significantly increases pore size and porosity (Meneghello et al., 2009), and the improved hydrophilicity should also decrease fouling potential (Howell et al., 1993).

The long-term aim is to produce a bioreactor containing biodegradable hollow fiber membranes that can be used by researchers and clinical technicians with expertise in cell culture, but not necessarily bioreactor design. This will require the provision of operating equations so that the end-users can set the pressures and feed flowrates to obtain the desired cell culture environment. The objective of the work presented in this paper is to define these operating equations for pure water by quantifying the membrane properties using a combined theoretical and experimental approach.

**Figure 1:**
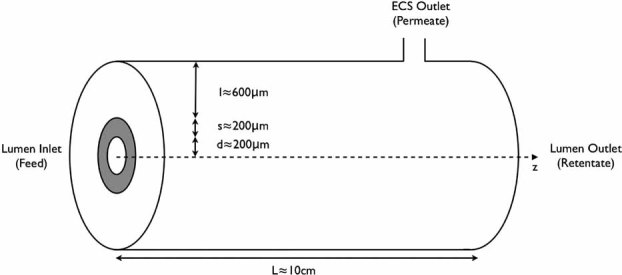
A schematic of a single fiber module of a hollow fiber bioreactor (not to scale). The module consists of a central lumen, surrounding porous wall (shaded gray), and extra-capillary space (ECS).

This paper considers pure water transport in a single fiber module of a hollow fiber bioreactor consisting of the central lumen, surrounding porous wall and extra-capillary space (ECS). A schematic of the setup is shown in Figure 1. Fluid is pumped into the lumen at a prescribed inlet flowrate, *Q*_*l*,in_ (mL/min); the lumen outlet pressure is controlled to maintain flow both through the lumen, and out to the ECS through the porous wall. The ECS outlet is fixed at atmospheric pressure. We are concerned with determining how the permeate to feed ratio (defined as the permeate flowrate as a fraction of the feed flowrate) depends on experimentally controlled parameters (e.g., the lumen inlet flowrate and lumen outlet pressure) and membrane properties. This will require fluid dynamical descriptions of how the fluid is transported in the lumen, membrane, and ECS.

Fluid transport in the lumen and ECS can be described using the Navier–Stokes' equations for an incompressible fluid of constant viscosity. The PVA–PLGA membrane is a synthetic porous structure with a reproducible mean pore diameter of 1.1 µm (which falls in the middle of the microfiltration pore diameter range 0.05–10 µm) and the overall porosity of the membrane is 0.77 (Meneghello et al., 2009). We can therefore use Darcy's law (Darcy, 1856) to describe fluid transport through the membrane; this says that the fluid velocity in the membrane is proportional to the pressure gradient across it, and the constant of proportionality is the ratio *k*/*µ*, where *k* is the membrane permeability, and *µ* is the fluid viscosity. The permeability *k* is unknown for our membranes, and is determined as part of the study. Typical values of *k* for different membrane types are difficult to isolate in the literature. This is primarily because few authors characterize membranes in terms of their hydrodynamic properties, but rather their solute or protein transport properties (e.g., they link membrane pore size or molecular weight cutoff to the permeability of a membrane to a particular protein). In addition, many engineering approaches lump together fluid flow and membrane properties rather than examining each of these in isolation. For example, the ratio of the transmembrane fluid flux to the difference between the lumen inlet and ECS outlet pressures depends on the fluid properties (the viscosity) as well as the system geometry and membrane properties. It should not, therefore, be regarded as a inherent membrane property.

When a free fluid flows past a permeable boundary (such as a membrane), a boundary layer develops in which the tangential velocity of the fluid on the membrane does not vanish. This phenomenon is called slip, and it is described by the parameter *α*. Beavers and Joseph (1967) were the first to consider the appropriate form of the boundary condition for the tangential component of the velocity when a Newtonian fluid flows over a porous interface by conducting pioneering experiments based on a 2D flow in a channel over a naturally permeable block, under an imposed pressure gradient. They concluded that a free fluid in contact with a porous medium flows faster than a fluid in contact with a completely solid surface, and a small boundary layer develops near the interface within which viscous shear forces are important and the tangential velocity of the fluid does not vanish. Saffman justified the Beavers and Joseph boundary condition theoretically in his paper (Saffman, 1971) by using a statistical approach to extend Darcy's law to non-homogeneous porous medium. Jones extended their result to curved boundaries and non-tangential flows (Jones, 1973).

In general, previous modeling approaches for HFB for tissue engineering applications have been motivated by understanding solute (e.g., oxygen, glucose) and protein distribution in a module. The mass transport models are superimposed on mathematical models for fluid transport, and have mainly focussed on the analysis of a single fiber unit (called the Krogh cylinder) assumed to be representative of the whole reactor (Abdullah and Das, 2007; Apelblat et al., 1974; Kelsey et al., 1990; Ye et al., 2006). Most approaches neglect convective effects (except in the lumen), and assume only radial diffusive transport of substrate in the membrane and ECS, with chemical reaction taking place in the latter.

One of the earliest examples of the Krogh cylinder approach is (Apelblat et al., 1974), where the authors use coupled Navier–Stokes equations (fiber lumen) and Darcy's law (membrane wall and ECS) to describe transport. Darcy's law was replaced with Navier–Stokes' equations to describe cross-flow filtration when there are no cells in the ECS (Kelsey et al., 1990). Bruining (1989) presented a general description of the hydrodynamics in hollow-fiber devices. The scope of his analysis included different modes of operation (e.g., closed-shell, continuous open-shell, suction of permeate, dead-end filtration) corresponding to various applications of HFBs. Starting from mass and momentum balance equations, Bruining obtained expressions for the hydrostatic pressure and bypass (fraction of fluid passing through the ECS). However, Bruining's simple analysis provides no information on local velocity profiles. A review of Krogh cylinder models for mass transport in HFBs for cell culture is presented by Brotherton and Chau (1996).

Two further Krogh cylinder approaches modeled the ECS as a porous medium to mimic a densely packed cell population (Abdullah and Das, 2007; Ye et al., 2006). The impact of multi-component interactions are also considered by using the Maxwell–Stefan equations to describe diffusion (Abdullah and Das, 2007). Numerical solutions using finite element methods are used to investigate the dependence of the nutrient concentration profiles on the cell density, medium flowrate, cellular matrix thickness, etc.

Finally, Labecki et al. (1995) moved away from the Krogh cylinder approach by developing a new porous medium model to capture the properties of a fiber bundle. The lumen and ECS are treated as two interpenetrating porous regions, with the membrane appearing through a source/sink term in the lumen and ECS equations. Macroscopic flow properties were investigated by solving the coupled partial differential equation model under different modes of operation. The impact of membrane wetting was also included by modifying the source/sink terms.

The flow problem that we consider here differs from these previous studies. First of all, in our setup the lumen outlet pressure is controlled to ensure fluid flow both through the lumen, and through the membrane and ECS. Therefore, fluid flow in the membrane and ECS must not be neglected. Secondly, we proceed with an analytical rather than numerical approach to analyze the fluid transport equations. This elucidates the underlying fluid dynamics, and enables analytical expressions for the fluid flowrates to be determined that could be readily applied to new situations. This approach also enables operating equations to be identified that are specific to our HFB setup.

Indeed, the aim of this study is to define operating conditions that facilitate bioreactor use for cell culture applications, by using a combined theoretical and experimental approach. In the Materials and Methods: Theory Section, a theoretical model is derived that describes the transport of pure water in the lumen, membrane wall, and ECS of a single fiber module, to represent a Krogh cylinder sub-unit. This model is reduced under radial symmetry, and by using lubrication theory (see the Supplementary Material Section 1), to provide equations for the retentate and permeate flowrates in terms of experimentally controlled parameters together with two unknowns: the membrane permeability, *k*, and slip, *α*. In the Materials and Methods: Experimental: Hollow Fibre Module Set-Up Section, the experimental procedure used to collect water flowrate data is outlined. In the Results and Discussion Section, the theoretical and experimental results are compared to determine *k* and *α*, to validate the models, and to determine the significance of slip. Additionally, an operating equation is determined that relates the permeate to feed ratio, *c*, to experimentally controlled parameters (specifically the lumen inlet flowrate, lumen outlet pressure, and ECS outlet pressure, together with parameters that capture the membrane permeability and geometry). Two worked examples are presented in the Results and Discussion: Worked Examples of the Operating Equations Section to demonstrate how this operating equation can be used by tissue engineers to setup their bioreactor. Finally, the conclusions are presented in the Conclusion Section.

## Materials and Methods

### Theory

#### Full System

Let *z* be the axial direction down the lumen, starting at the lumen inlet (*z* = 0) with the lumen outlet denoted by *z* = *L*. We denote the radius of the lumen by *d*, the depth of the lumen wall by *s* and the depth of the ECS by *l*. Typically, *L* ≍ 10 cm, *d* ≍ 200 µm, *s* ≍ 200 µm, and *l* ≍600 µm.

We denote the fluid velocity and pressure by **u** and *p*, with subscripts *l*, *w*, and *e* denoting the lumen, lumen wall, and ECS, respectively. We describe fluid flow in both the lumen and ECS by the Navier–Stokes' equations for an incompressible fluid of constant viscosity. For an introduction to the fluid dynamic equations used here, refer by Kay and Nedderman (1974). The equations in the lumen and ECS are

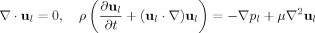
2

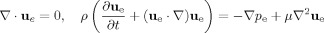
3
where *ρ* and *µ* are the fluid density and viscosity, respectively. The left-hand equations represent conservation of mass, and the right-hand equations represent conservation of momentum, as a balance of inertia, pressure, and viscous forces.

The lumen wall is a synthetic porous structure composed of a blend of PVA and PLGA (in the ratio 1:3) (Ellis and Chaudhuri, 2007). We assume that the wall is isotropic and the permeability is constant, so that a simplified version of Darcy's Law with effective membrane permeability *k* (units m^2^) may be used. This assumption is valid as we are interested in the averaged properties of the membrane, rather than the fluid dynamics at the pore-scale. This membrane permeability *k* is unknown, and will be determined from experimental data. For an introduction to flow in porous media, refer to de Nevers (1991). In the lumen wall,

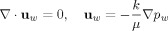
4
where the left-hand equation again represents conservation of mass, and the right-hand equation is a constitutive relationship that relates the fluid velocity in the wall to the pressure gradient across it, through the wall permeability.

On the lumen/wall and wall/ECS boundaries we prescribe conservation of fluid flow and continuity of pressure, so that

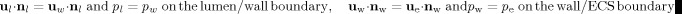
5
where ***n***_*l*_ and ***n***_w_ are the unit outward pointing normals to the lumen/wall and wall/ECS boundaries, respectively. Finally, we impose boundary conditions to account for slip at the membrane surface, as described in the Introduction Section. The boundary conditions on the lumen/wall and wall/ECS boundaries are

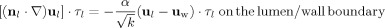
6

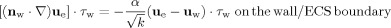
7
where the left-hand terms represent viscous shear, and the right-hand terms the difference in tangential velocities that it induces. Here **τ**_*l*_ and **τ**_*w*_ are the unit tangential vectors (along the fiber wall in the axial *z*–direction) to their respective surfaces, and *α* is a dimensionless slip constant that depends on the surface properties. This parameter *α* is unknown for our hollow fiber membranes, and must be determined from experimental data. The width of the boundary layer region (where the tangential velocity component does not vanish) is 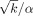.

In addition, there is no flux of fluid out of the ECS at *z* = 0 and *z* = *L* (the ECS is glued at the ends), therefore

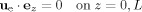
8
where **e**_*z*_ is the unit vector in the *z*-direction. Finally, we treat the entire outer ECS boundary as the outlet and impose pressure and velocity boundary conditions on this surface. These are

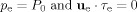
9
where *P*_0_ is atmospheric pressure. There are two modeling assumptions underlying these boundary conditions: first of all, we have not modeled the ECS outlet explicitly (but rather treated the entire outer ECS boundary as the outlet), and in the second condition we have imposed no axial component of flow on this outlet (as we are expecting radial flow to dominate here). It is anticipated that neither of these will significantly impact the validity of the model as the bulk of the radial pressure drop from the lumen to the ECS will occur across the membrane wall. This is confirmed a priori, when the model predictions are compared against experimental data.

Experimentally, we prescribe the volumetric flowrate of fluid into the lumen

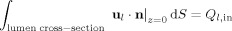
10
where the integral is over the cross-section of the lumen at *z* = 0. We also prescribe the pressure at the lumen outlet (maintained using a clamp to provide the required back pressure to obtain the desired ratio of permeate to retentate flowrates),

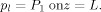
11

The volumetric flowrate of fluid leaving the lumen and ECS can also be measured experimentally; these are given by

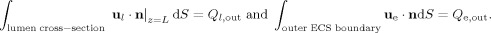
12

Conservation of fluid mass means that the inlet and outlet flowrates must satisfy

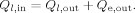
13

#### Reduced System

The system of equations given by (2)–(13) can be simplified by assuming that the set-up is radially symmetric, and by exploiting the small aspect ratio of the fibers. The Reynolds number, Re, describes the relative importance of inertial and viscous forces in the fluid. Based on the axial flow in the lumen, Re = *UL*/*ν*, where *U* is the lumen flow velocity and *ν* = 1.004 m^2^ s^−1^ is the kinematic viscosity of water (at 20°C). The lumen inlet flowrate, *Q*_*l*,in_ is fixed by a pump at ≍3 mL/min = 5 × 10^−8^ m^3^ s^−1^. The cross-sectional area of the lumen is π*d*^2^ ≍ 12.6 × 10^−8^ m^2^. Therefore, a typical lumen inlet velocity is *U* = 0.40 m s^−1^, and Re = *UL*/*ν* ≍ 4 × 10^4^. Defining the aspect ratio of the fiber, *ε*, by *ε* = *d*/*L* = 2 × 10^−3^, then the reduced Reynolds number, *ε*^2^Re ≍ 1.6 × 10^−1^. This reduced Reynolds number characterizes the fluid flow regime by taking account of both the balance of inertial and viscous forces, and the small aspect ratio of a fiber. Given that *ε*^2^Re is small, it is appropriate to consider lubrication theory (valid when *ε* << 1 and *ε*^2^Re << 1) to simplify the full set of equations (Ockendon and Ockendon, 1995). For the mathematical detail of this reduction, refer to the Supplementary Material Section 1.

For ease of notation, we define dimensionless parameters by

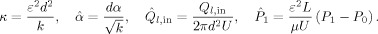
14

These parameters capture the key physical features of the system; *κ* represents the permeability of the membrane wall, 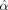 the importance of slip versus permeability on the membrane surface, 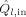 the fluid flowrate into the lumen inlet, and 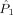 the difference between the lumen outlet and ECS outlet pressures. We normalize velocities in the system to the lumen inlet velocity by choosing *U* = *Q*_*l*,in_/(2π*d*^2^) so that 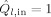 throughout and *Q*_*l*,out_ and *Q*_e,out_ will be less than one.

The analysis described above results in the following expressions to obtain values for the lumen and ECS outlet (i.e., retentate and permeate, respectively) flowrates:

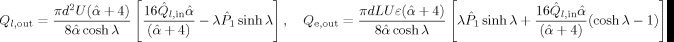
15
where the coefficient λ is given by

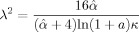
16
and *a* = *s*/*d* is the ratio of the wall depth to the lumen radius. Two parameters are unknown: the membrane permeability, *k*, and the slip parameter, *α*. The remaining parameters in (15) are properties of the setup, or are experimentally fixed: these are summarized in Table I.

**Table I:** A table of the parameter values required to determine the retentate and permeate flowrates from Equation 15.

Parameter	Description	Value	Units
Geometrical properties of the setup			
*d*	Radius of the lumen	200	µm
*s*	Depth of the membrane wall	200	µm
*l*	Depth of the ECS	600	µm
*L*	Length of the fiber	10	cm
Experimentally imposed quantities			
*Q*_*l*,in_	Lumen inlet flowrate	^*^	mL/min
*P*_0_	ECS outlet pressure (atmospheric)	14.69	psia
*P*_1_	Lumen outlet pressure	^*^	psia
Unknown membrane characteristics			
*k*	Membrane permeability	Unknown	m^2^
α	Membrane slip parameter	Unknown	dimensionless
Modeling parameters			
ε	Aspect ratio of a fiber	2 × 10^−3^	dimensionless
*a*	Dimensionless depth of the membrane wall	1	dimensionless
*b*	Dimensionless depth of the ECS	3	dimensionless
*κ*	Dimensionless membrane permeability [see (14)]	Unknown	dimensionless
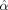	Importance of slip versus permeability on the membrane surface [see (14)]	Unknown	dimensionless
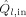	Dimensionless fluid flowrate into the lumen [see (14)]	1	dimensionless
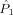	Dimensionless lumen exit pressure [see (14)]	^*^	dimensionless
*λ*	Dimensionless parameter defined by (16)	^*^	dimensionless

These are made up of parameters that are fixed by the setup (i.e., geometrical parameters), parameters that are experimentally imposed, the unknown membrane characteristics, and dimensionless parameters identified by the modeling. ^*^ represents quantities which are fixed experimentally, but which we vary from experiment to experiment to complete the model parameterization and validation.

Next, we use a single hollow fiber module setup to determine both *k* and *α*, by fitting experimentally measured values of the retentate and permeate flowrates against Equation 15 for a fixed value of the lumen inlet flowrate *Q*_*l*,in_. The model is then validated by comparing the model predictions of *Q*_*l*,out_ and *Q*_e,out_ against experimentally measured values for a different lumen inlet flowrate *Q*_*l*,in_.

### Experimental: Hollow Fiber Module Set-Up

PVA–PLGA hollow fiber membranes were prepared from a spinning dope of 1:3:16 PVA:PLGA:NMP, and were fabricated in-house as previously described (Meneghello et al., 2009). 75:25 *P*_DL_LA:PGA PLGA (756 S) was purchased from Resomer (Boehringer Ingelheim, Ingelheim, Germany), PVA from Sigma–Aldrich (Poole, UK) (8–10 kDa), NMP (extra pure 127630025; Acros Organics, Loughborough, UK) was used as the solvent and deionized water used as the non-solvent. The fibers had a mean pore diameter of 1.1 µm, lumen diameter of 400 µm, and wall thickness of 200 µm.

A single fiber was fixed into a glass module, with two side ports 1 cm from the ends (one of which was closed off), using Araldite Precision epoxy resin (Bostik Ltd, Leicester, UK). Distilled water was pumped though the fiber lumen using a Masterßex L/S Digital Standard Drive peristaltic pump. A crossflow configuration was used and a clamp on the lumen outlet was used to control the relative pressure gradients down the lumen and across the membrane. The membrane was wetted for 2 h, and the system left to equilibrate for 5 min after each change in inlet flowrate. Pressures were measured using pressure gauges (15 psi ±0.5) (Ashcroft) on the lumen inlet and outlet, and the retentate and permeate flowrates were measured using timed collection at 1 min intervals using Mettler Toledo PG503-5 mass scales, accurate to ±0.005 g.

## Results and Discussion

### Data Fitting to Determine the Membrane Permeability and Slip Parameter

The membrane permeability, *k* (m^2^) and slip parameter, *α* (dimensionless) are determined by comparing the theoretical retentate and permeate flowrate expressions given by (15) with water flowrate data for a lumen inlet flowrate of 3.13 mL/min. The water mass of retentate and permeate, and lumen outlet pressure were recorded at 1 min intervals for 6 min; the raw data is given in the Supplementary Material Section 2. Based on this raw data, values of *Q*_*l*,out_ and *Q*_e,out_ are calculated by dividing the mass values (kg) by the product of the time interval (60 s) and water density (998.21 kg m^−3^ at 20°C). Further, the value of 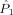 at each time was calculated using the relationship in (14). The values of *Q*_*l*,out_, *Q*_*e*,out_, and 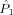 are summarized in Table II.

**Table II:** The calculated values of *Q*_*l*,out_, *Q*_e,out_ and 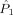 based on the raw data for an inlet flowrate of 3.13 mL/min (see the Supplementary Material Section 2).

Time (min)	*Q*_*l*,out_ (10^−8^ m^3^ s^−1^)	*Q*_e,out_ (10^−9^ m^3^ s^−1^)	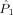 (dimensionless)
1	4.85	3.50	17.13
2	4.88	3.67	17.13
3	4.83	3.83	17.13
4	4.82	3.00	17.13
5	4.88	3.67	17.13
6	4.90	3.67	17.13

These data were inputted into Equation 15 at each time point; the remaining parameters are given in Table I, together with *U* = 0.4161 m s^−1^. Numerically solving Equation 15 using these values determines *κ* and 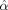 at each time point; the permeability *k* and slip parameter *α* can then be determined from the relationships

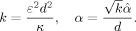
17

The mean values of *κ* and 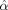 are 692.03 and 97.82, respectively, corresponding to mean values of *k* and *α* of 2.31 × 10^−16^ m^2^ and 7.44 × 10^−3^, respectively. The mean relative errors (defined as the positive difference between the experimental mass measurement and theoretical mass prediction, normalized by the experimental value, for each data point) are 0.68% and 0.71% for the retentate and permeate, respectively.

### Model Validation

We validate the model, together with the mean values of *k* and *α* determined in the Data Fitting to Determine the Membrane Permeability and Slip Parameter Section, by comparing the theoretical predictions of retentate and permeate flowrates against experimentally measured values for a new lumen inlet flowrate of 4.66 mL/min. Equation 15 was used to predict the cumulative mass of water collected for this new inlet flowrate; this was compared to experimental data (raw data is presented in the Supplementary Material Section 2). The same parameter values as in the Data Fitting to Determine the Membrane Permeability and Slip Parameter Section were used, although now *U* = 0.6193 m s^−1^, and the mean values of *k* and *α* were inputted.

**Figure 2:**
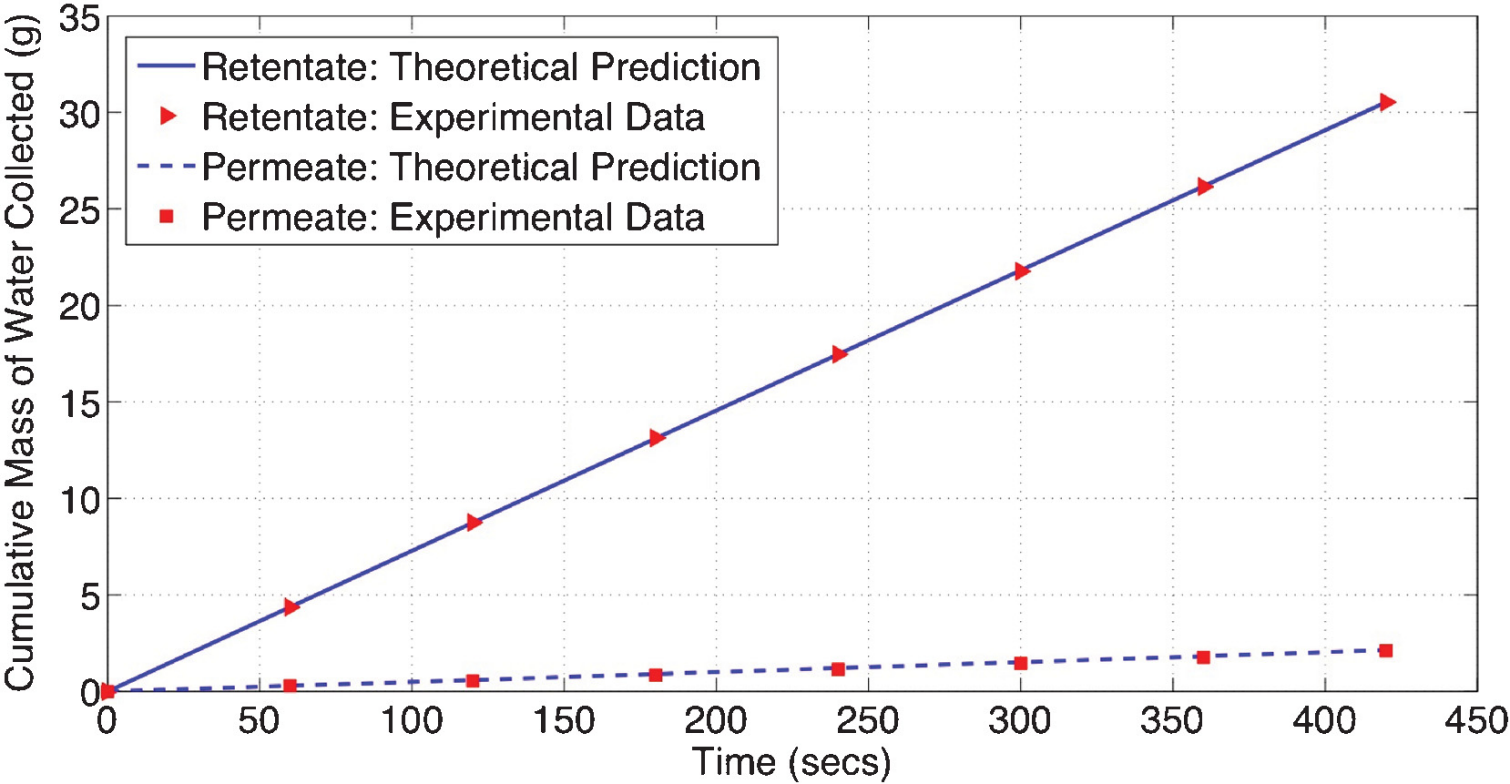
Comparison of experimental and theoretical predictions for the cumulative mass of retentate and permeate collected, when *Q_l_*_,in_ = 4.66 mL/min.

A comparison of the theoretical predictions and experimental data is given in Figure 2. The agreement between the two is excellent; the mean relative errors are 0.62% and 0.54% for the retentate and permeate, respectively. The models can now be used to predict the velocity and pressure distributions in new regimes.

### Slip Analysis

Next, we investigate the sensitivity of the retentate and permeate flowrate expressions to the slip parameter *α*, to determine whether slip at the membrane surface significantly impacts determination of the average properties of the membrane. As explained previously, a free fluid flows faster past a permeable boundary than it does past an impermeable boundary, and, in fact, a boundary layer develops in which the tangential velocity of the fluid on the membrane does not vanish. This phenomenon is called slip, and it is described by the parameter *α*. Here, two boundary layers will develop in the membrane: one on the lumen/wall boundary, and another on the wall/ECS boundary. Both of these boundary layers will have width of size 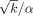 (see Eqs. 6 and 7). Using the mean values of *k* and *α* determined in the Data Fitting to Determine the Membrane Permeability and Slip Parameter Section, this width is 2.047 µm, and so the total width of boundary layers is 2.05% of the membrane wall thickness. This is only a small percentage, and so it seems highly unlikely that slip has a significant effect on the average properties on the membrane.

**Figure 3:**
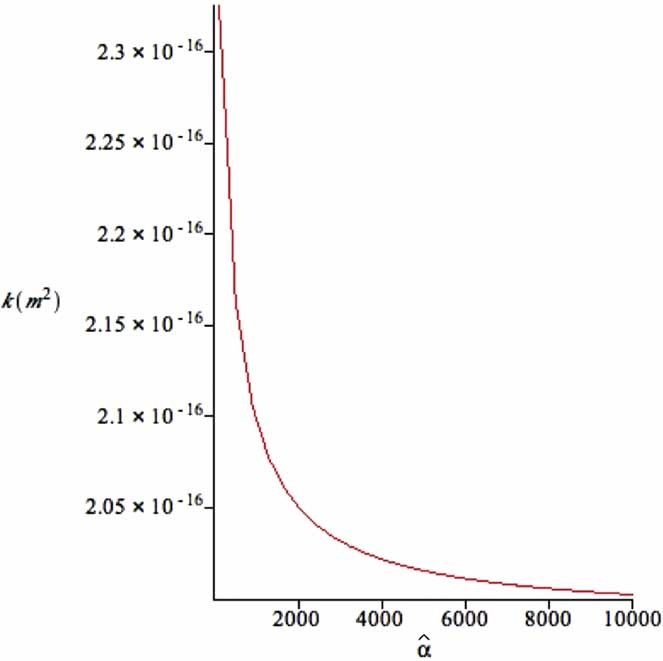
A graph of *κ* against 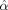, calculated using equation the retentate flowrate expression in (15).

This conclusion is backed up by Figure 3, which shows a plot of the membrane permeability *k* (m^2^) against the dimensionless slip parameter 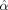, calculated using the retentate flowrate equation given in (15) (using the *Q*_*l*,out_, *Q*_e,out_ and 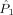 values at time =3 min). Here, *k* varies between 2.00 × 10^−16^ and 2.35 × 10^−16^ m^2^, whilst 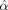 varies between 0 and 10^4^.

To test the hypothesis that membrane slip is not a significant effect, we compare the retentate and permeate flowrate predictions under no slip to the experimentally measured values. No slip is equivalent to taking the limit as 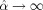 in the modeling equations, and so the retentate and permeate flowrate equations are now given by

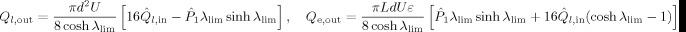
18
where *λ*_lim_ is defined by

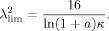
19

**Figure 4:**
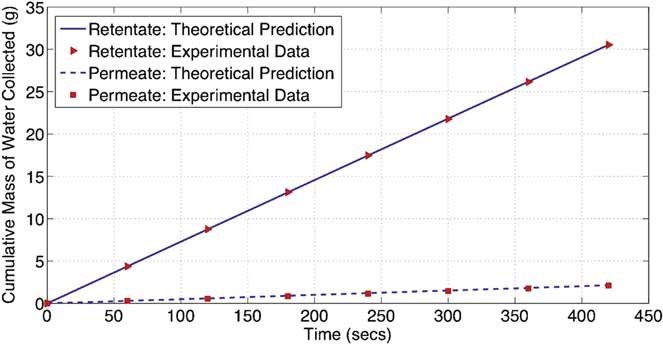
Comparison of experimental data and no slip theoretical predictions when *Q_l_*_,in_ = 4.66 mL/min.

Firstly, we fit the experimental data for *Q*_*l*,in_ = 3.13 mL/min against the retentate and permeate flowrate expressions (18), to determine the new value of the membrane permeability *k* under the no slip assumption; this yields a mean value of *k* of 1.86 × 10^−16^ m^2^ [using either expression in (18)]. Next, we compare the theoretical predictions of retentate and permeate flowrates given by (18) (using this new mean value of *k*) against the experimental data for *Q*_*l*,in_ = 4.66 mL/min; this is shown in Figure 4. The mean relative errors are 0.57% and 0.35% for the retentate and permeate, respectively, indicating an excellent agreement between theoretical predictions and experimental values. These mean relative errors are, in fact, smaller than when slip was included (this is an artefact of using mean values of *k* and *α*, rather than the values calculated at each time point). Therefore, we conclude that slip is an insignificant effect when dealing with the averaged properties of PVA–PLGA microfiltration membranes.

### Permeation to Feed Ratio

Next, we use the parameterized and validated no-slip model to establish operating conditions that enable the feed flowrate and retentate pressure to be controlled to provide a specific permeation to feed ratio. We let

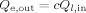
20
so that the permeate flowrate is *c*-times the feed flowrate, where 0 < *c* < 1.We refer to *c* as the “permeate to feed ratio.” Combining the relationship for *Q*_e,out_ in (18) with (20) gives an expression for 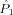 in terms of *c* and experimentally fixed parameters,

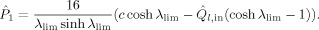
21

Using the mean value of *k* calculated in Slip Analysis Section, Equation 21 now gives a relationship between 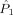 and *c*. Any change in 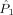 can be achieved by varying either the lumen inlet flowrate, *Q*_*l*,in_, or the lumen outlet pressure, *P*_1_, because 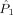 is related to these quantities through

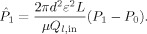
22

Equations 21 and 22 permit operating conditions to be specified to achieve a particular value for the permeate to feed ratio, *c*. The operating equation is given by

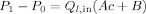
23
where

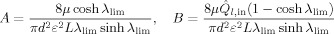
24
are constants that depend on the membrane permeability and geometry.

**Figure 5:**
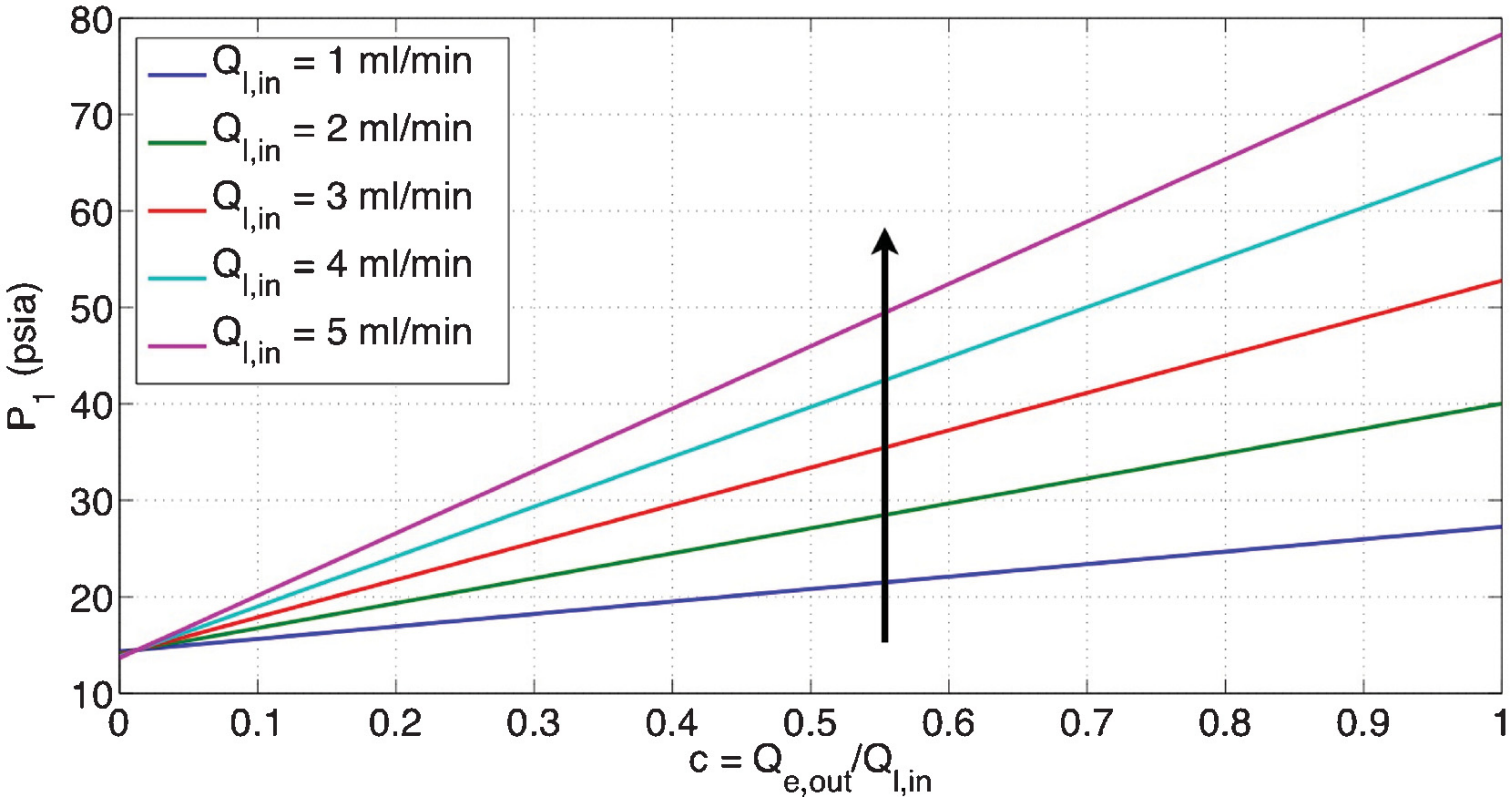
A plot of *P*_1_ as a function of *c* = *Q*_e,out_/*Q_l_*_,in_, with the inlet flowrate *Q_l_*_,in_ fixed. The arrow shows the direction of *Q_l_*_,in_ increasing.

A plot of *P*_1_ as a function of the ratio *c* (for fixed *Q*_*l*,in_ = 1, 2, 3, 4, 5 mL/min) is shown in Figure 5. As would be anticipated, as the permeate to feed ratio increases, the lumen outlet pressure *P*_1_ required to maintain this ratio increases (for a fixed inlet flowrate). As the inlet flowrate (*Q*_*l*,in_) increases, a larger lumen outlet pressure *P*_1_ is required to maintain the same permeate to feed ratio, *c*.

In particular, experimentally the lumen outlet pressure must always be greater than atmospheric pressure, so that *P*_1_ > *P*_0_. Equation 23 gives a minimum value for *c* for which this is true; this is given by

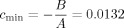
25
independent of the value of the lumen inlet flowrate, *Q*_*l*,in_. This can be seen in Figure 5, where each of the lines pass through *P*_1_ = 14.5 = *P*_0_.

**Figure 6:**
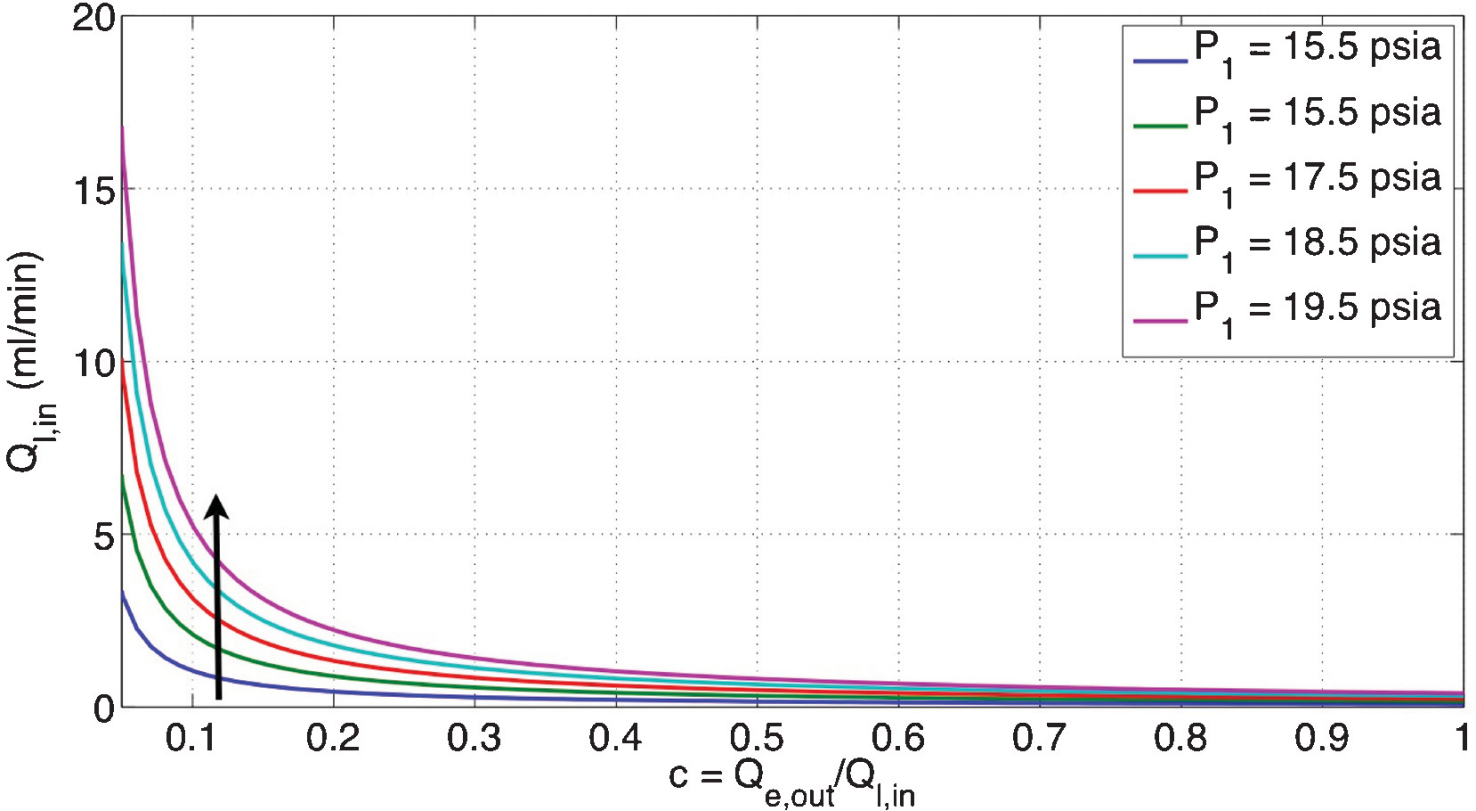
A plot of *Q_l_*_,in_ as a function of *c* = *Q*_e,out_/*Q_l_*_,in_, with the lumen outlet pressure *P*_1_ fixed. The arrow shows the direction of *P*_1_ increasing.

The behavior exhibited in Figure 5 is mirrored in Figure 6, which shows a plot of *Q*_*l*,in_ as a function of *c* (for fixed *P*_1_ = 1, 2, 3, 4, 5 psi). As the permeate to feed ratio *c* increases, the inlet flowrate *Q*_*l*,in_ required to maintain this ratio decreases (for a fixed lumen outlet pressure). As the lumen outlet pressure, *P*_1_, increases, a larger inlet flowrate, *Q*_*l*,in_, is required to maintain the same permeate to feed ratio, *c*.

### Worked Examples of the Operating Equations

Let us consider two examples that might be encountered by a tissue engineer when setting up a hollow fiber bioreactor system. For the sake of argument, let us assume that the system under consideration has the same geometrical properties as the one in this paper, so that the lumen radius, depth of the membrane wall and length of the fiber are given by *d* = 200 µm, *s* = 200 µm, *L* = 10 cm, respectively. From the analysis in this paper, the membrane permeability is *k* = 1.86 × 10^−16^ m^2^, and we consider the flow of water with dynamic viscosity *µ* = 8.90 × 10^−4^ N s/m^2^. On this basis, we can calculate the components *A* and *B* of the operating equation, Equation 23, that depend purely on the membrane permeability and geometry. The components *A* and *B* are given by Equation 24

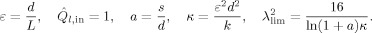
26

Therefore, in this case we have *ε* = 2 × 10^−3^, *α* = 1, *κ* = 861.93, *λ*_lim_ = 0.1636, together with *A* = 5.34 × 10^12^ kg m^−4^ s^−1^, and *B* = −1.41 × 10^11^ kg m^−1^ s^−1^. Clearly *A* and *B* would take different values for membranes with different permeability and geometrical properties.

Worked Example 1 A tissue engineer wants to supply fluid to the lumen at a flowrate of 2 mL/min, and to achieve a permeation to feed ratio of 0.2. What pressure should they apply on the lumen outlet?

The lumen outlet pressure, *P*_1_, required to achieve this can be calculated from the operating equation, Equation 23. We have a feed flowrate, *Q*_*l*,in_ = 2 mL/min; in SI units this is *Q*_*l*,in_ = 3.34 × 10^−8^ m^3^ s^−1^. The permeation to feed ratio, *c* = 0.2, and atmospheric pressure, *P*_0_ = 10^5^ Pa. Therefore,

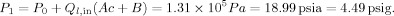
27

Worked Example 2 A tissue engineers wants to fix the lumen outlet pressure at 30 psia (=15.50 psig), and to achieve a permeation to feed ratio of 0.5. What feed flowrate should they impose?

The feed flowrate, *Q*_*l*,in_, required to achieve this can be calculated from the operating equation, Equation 23. We have a lumen outlet pressure, *P*_1_ = 30 psia; in SI units this is *P*_1_ = 206,850 Pa. The permeation to feed ratio, *c* = 0.5, and atmospheric pressure, *P*_0_ = 10^5^ Pa. Therefore,

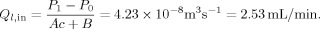
28

## Conclusions

In this paper, we have established a theoretical model that captures the key physical components of fluid flow in a single module of a HFB, including both the permeability of the porous membrane, and slip at the membrane surface. We have combined the theoretical approach with experimental studies of pure water transport to characterize the membrane permeability and slip. Analysis of the width of the boundary layer in which slip effects are important, together with the sensitivity of the retentate and permeate flowrate equations to the slip parameter, showed that slip was not significant when considering averaged membrane properties.

The models were reduced under the assumption of no-slip at the membrane surface. Comparing with flowrate data for a lumen inlet flowrate of 3.13 mL/min determined the mean membrane permeability to be *k* = 1.86 × 10^−16^ m^2^; the theoretical predictions were then validated against flowrate data for 4.66 mL/min (the mean relative errors for the retentate and permeate flowrates were 0.57% and 0.35% respectively).

Finally, the parameterized and validated models were used to predict operating regions under which the lumen inlet velocity, *Q*_*l*,in_, and lumen outlet pressure, *P*_1_, are controlled to provided a fixed permeate to feed ratio, *c*. The operating equation is given by

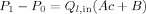
29
where

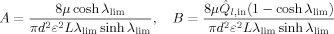
30
are constants that depend on the membrane permeability and geometry. Equation 29 can now be implemented by end-users to set the pressures and feed flowrates to obtain the permeate to feed ratio required for the desired cell culture environment.

Further extensions to this work include modeling a porous material in the ECS to mimic growing tissue, and mass transport modeling for key nutrients (such as oxygen and glucose) and proteins (such as growth factors). The transport modeling will include fouling by media components, the next stage in the study, which would correspond to a time-dependent membrane permeability due to pore blockage/constriction/cake formation/a combination. A further extension of this will account for additional time dependent properties due to degradation of the membrane.

## Nomenclature

*d*: radius of the lumen (m)
*s*: depth of the lumen wall (m)
*l*: depth of the ECS (m)
*L*: length of a single module (m)
*z*: axial length coordinate down the lumen
*r*: radial coordinate
**u**: fluid velocity vector (m s^−1^)
*p*: fluid pressure (Pa)
*ρ*: fluid density (kg m^−3^)
*µ*: fluid dynamic viscosity (Pa s)
*k*: permeability of the lumen wall (m^2^)
**n**: unit outward pointing normal vector to relevant surface
**τ**: unit tangent vector to relevant surface
*P*_1_: lumen outlet pressure (Pa or psi for experimental data)
*P*_0_: ECS outlet pressure (Pa, or psi for experimental data)
*Q*_*l*,in_: volumetric flowrate of fluid into the lumen inlet (m^3^ s^−1^ or mL min^−1^ for experimental data)
*Q*_*l*,out_: volumetric flowrate of fluid out of the lumen outlet (m^3^ s^−1^ or mL min^−1^ for experimental data)
*Q*_e,out_: volumetric flowrate of fluid out of the ECS outlet (m^3^ s^−1^ or mL min^−1^ for experimental data)
*U*: velocity scale (m s^−1^)
*P*: pressure scale (Pa)
*ε*: aspect ratio of the lumen
Re: Reynolds number based on the fiber length
*a*: dimensionless depth of the lumen wall
*b*: dimensionless depth of the ECS
α: Beaver's–Joseph slip coefficient
κ: Dimensionless permeability of the lumen wall
dimensionless parameter representing the importance of slip versus permeability on the membrane surface
dimensionless flowrate of fluid into the lumen inlet
dimensionless difference between the lumen outlet and ECS outlet pressures
*c*: permeate to feed ratio
